# Effects of breccia and water contents on the mechanical properties of fault-core-zone materials

**DOI:** 10.1038/s41598-022-10995-2

**Published:** 2022-04-30

**Authors:** Hyun-Seok Yun, Seong-Woo Moon, Yong-Seok Seo

**Affiliations:** 1 Civil and Architectural, Environmental Engineering Department, KEPCO Engineering & Construction, Gimcheon, 39660 Republic of Korea; 2 Department of Earth and Environmental Sciences, Chungbuk National University, Cheongju, 28644 Republic of Korea

**Keywords:** Solid Earth sciences, Engineering

## Abstract

Determining the mechanical properties of fault-core-zone materials is challenging because of the low strength of such materials, which affects field sampling, specimen preparation, and laboratory testing. We overcame this problem by preparing and testing mechanical properties of 132 artificial fault-core-zone specimens consisting of mixtures of breccia, sand, clay, and water. The unconfined compressive strength (UCS), elastic modulus (E), and penetration resistance value (PRV) of these fault-core-zone materials were measured, and the effects of breccia content and water content on mechanical properties were assessed. Results show that UCS is inversely proportional to breccia content and water content, and that E is inversely proportional to water content. Furthermore, the inverse relationship of UCS with water content varies with breccia content. UCS is proportional to both PRV and E, and the relationship for each varies with breccia content. High coefficients of determination (R^2^ = 0.62–0.88) between the parameters suggest that breccia content, water content, and PRV are potentially useful parameters for estimating the mechanical properties of fault core zones.

## Introduction

A fault core zone commonly includes fault gouge (or clay), cataclasite, breccia and fragmented country rock, and is a zone of intense deformation surrounded by a fault damage zone^1–10^. Fault core zones, also termed fault-slip zones, accommodate most of the displacement during faulting, causing the existing material to be crushed and altered^11^. As fault-core-zone material (hereinafter referred to as the “fault core”) has lower strength than the surrounding rock mass, it is classified as critical geomaterial that can cause geotechnical problems in rock engineering projects^12–16^. Therefore, it is very important to clearly understand and determine the physico-mechanical properties of fault core. However, to determine the mechanical properties of fault-core, high-quality samples are needed^17^. Unfortunately, the weakness of such materials and the alignment of constituent particles mean that sampling in the field and sample preparation for laboratory testing are challenging and often compromised or unsuccessful^18–20^. Kanji^21^ explained that it is difficult to test using typical sampling, site investigation, and rock mechanics equipment. Problems associated with fault core diversity and complexity as well as sampling, handling and preservation in connection with experimental work mean that geotechnical investigation is challenging. This can in turn lead to high project costs because they encourage engineers to apply conservative parameters in design and construction^14,21,22^.

Previous studies of the physico-mechanical properties of fault cores have focused mostly on analysis of the frictional behavior of clay-rich gouge^23–29^. In particular, the shear characteristics of fault core, such as coefficients of friction using shear tests^30–32^ and correlations between particle characteristics (e.g., size) and shear strength^33–37^, have been investigated more often than other properties (e.g., unconfined compressive strength (UCS) and elastic modulus (E)) because of the easier sampling and specimen preparation involved. On the other hand, to determine the mechanical properties (e.g., UCS and E) of fault core, high-quality samples are needed. Unfortunately, the weak bonding of fault means that sampling in the field and sample preparation for laboratory testing are challenging. For these reasons, previous analyses for UCS and E have limited on soft rocks (e.g., fault rocks, coarse pyroclastics, sheared serpentinites, and mélanges including gouge, clay, and breccia) which specimens shaping is possible on^16,38–43^.

To overcome the difficulties of sampling and preparing specimens from the fault core for conventional geomechanics tests, some studies have attempted to measure mechanical properties by either testing artificial specimens^20,44–46^, by using simpler tests and equipment^19,20,47–51^ and by the applications of statistical/modeling techniques such as regression analysis, fuzzy logic, and neural networks^52–58^. Measuring the physico-mechanical properties of artificial specimens makes it possible to control some parameter values and experimental conditions^46^, and previous studies using this approach have yielded meaningful proxies for the characterization of natural rocks^44,59^. Simple tests such as the needle penetration test (NPT), Schmidt hammer test, point load test, Shore hardness index, and block punch strength index all have the advantage of simple specimen preparation on natural samples compared with tests of UCS and E^19,20^. In addition, statistical and modeling methods have an advantage in estimating the mechanical properties using multiple factors. Although these various approaches and tests can provide reasonably reliable results and yield a better understanding of the physico-mechanical properties of soft materials, few studies have been conducted on fault cores.

In this study, we conducted laboratory tests on artificial specimens to investigate the physico-mechanical properties of fault core, natural examples of which are difficult to sample in the field. We prepared 132 artificial specimens comprising mixtures of different proportions of breccia, sand, clay, and water. Unconfined compression tests and penetration resistance tests were conducted on these artificial specimens, and their mechanical properties (UCS, E, and penetration resistance value (PRV)) were calculated. The data were analyzed to quantify the effects of breccia content and water content on mechanical properties as well as to establish relationships (correlations) between factors related to the mechanical properties of fault core.

## Preparation of artificial specimens

### Materials

**Figure 1:**
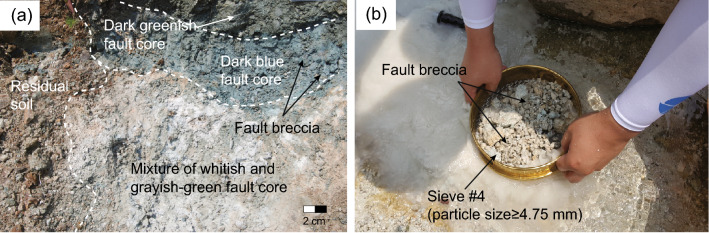
(**a**) Outcrop photograph of fault core sampled from the Ocheon Fault Zone for the manufacture of artificial specimens. (**b**) Breccia from the fault core zone classified using sieve #4 (≥ 4.75 mm).

Artificial specimens were made by mixing breccia, sand, and clay, which are the major components of fault core, with water. No bonding materials were added so as to more closely simulate natural fault material. The breccia used was obtained from the Ocheon Fault in southeastern Korea (Fig. 1a). The Ocheon Fault is the largest among the Ocheon Fault System composed of a number of NE or NNE-trending normal-slip and sinistral-normal oblique-slip faults, and is known to have led major tectonic activity with several crustal deformations during the Cenozoic^60,61^. In addition, this fault is the boundary between the Early Miocene Janggi and the Middle Miocene Pohang basins, SE Korea, and has a scissor fault geometry decreasing in vertical offset southwestward^62^. It was initially the northwestern border fault of the Janggi Basin, but it is interpreted that it was reactivated as the eastern border faults of the Pohang Basin with a sudden movement at about 17 Ma. The host bedrock of this fault zone is mainly granite, with lesser granodiorite and andesite. For this study, breccia was classified as particle sizes of ≥ 4.75 mm (sieve #4) after sampling numerous fault core materials along this fault (Fig. 1b). In geology, breccia size has been defined differently by various investigators, over a wide range from 0.1 to 5.0 mm^63–69^. Here, the size was based on the internationally recognized unified soil classification system (USCS) by American Society for Testing and Materials (ASTM 2487-17)^70^, which is commonly used in soil mechanics and rock engineering projects. Sand was classified as falling between sieves #4 and #200 (4.75 mm ≥ particle size ≥  0.075 mm).

**Figure 2:**
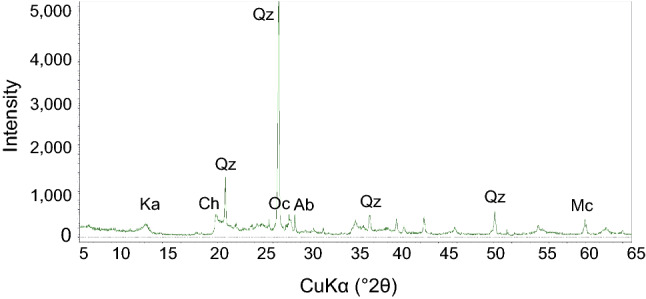
Results of XRD analysis of the clay material used in the manufacture of artificial specimens. Mineral abbreviations: albite (Ab), chlorite (Ch), kaolinite (Ka), microcline (Mc), orthoclase (Oc), and quartz (Qz).

**Figure 3:**
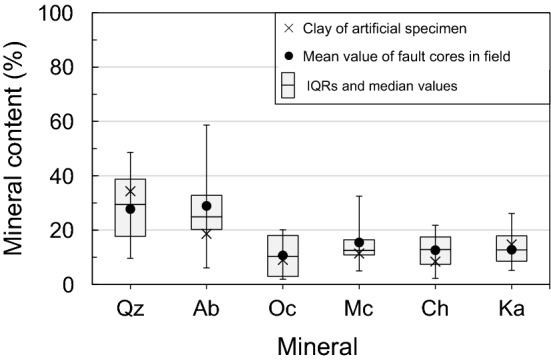
Box plots showing the mineral contents of 40 fault-core-zone clays sampled from the field and mineral contents of the clay material used for artificial specimen manufacture superimposed on the box plots. The boxes are inter-quartile ranges (IQRs). Mineral abbreviations: albite (Ab), chlorite (Ch), kaolinite (Ka), microcline (Mc), orthoclase (Oc), and quartz (Qz).

Clay (< 0.075 mm) is not always present in large volumes in outcrops of fault materials, and it is difficult to extract only clay from fault core zone. Accordingly, for the present study, ceramics clay was used as the clay material in the manufacture of artificial specimens. The major mineral compositions of the clay material used, as analyzed by X-ray diffraction (XRD), are quartz (34.3 vol.%), albite (18.6 vol.%), kaolinite (14.7 vol.%), and microcline (11.4 vol.%) (Fig. 2, Table 1). Figure 3 shows box-and-whisker plots comparing the mineral contents of the clay material used and 40 fault-core-zone clays (sampled from outcrops of the several fault zones in southeastern Korea). The mineral contents of the clay material used in this study lie mostly within the inter-quartile range of the natural fault-core-zone clays, and there is no statistically significant difference between the mean or median mineral contents of the clay material and the fault-core-zone clays. Consequently, the mineralogy of the clay material used in the artificial specimens was highly similar to that of fault-core-zone clays sampled from the field.

**Table 1:** Mineral contents of the clay used for manufacturing artificial specimens, as determined by XRD analysis.

Mineral	Quartz	Albite	Kaolinite	Microcline	Orthoclase	Chlorite	Rest
Content (vol.%)	34.3	18.6	14.7	11.4	9.0	8.4	3.6

### Determination of mixture component ratios

The mixing ratios of materials in the artificial specimens were determined by sampling and particle-size analysis of natural fault cores (a total of 96 material samples). The sieve test method of ASTM D422-63^71^ was applied simultaneously with the soil-washing test method of ASTM D1140-17^72^ to clearly separate the components in the natural fault core.

**Figure 4:**
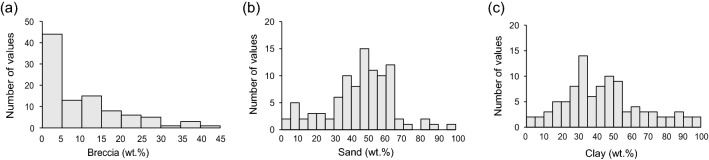
Histograms of (**a**) breccia, (**b**) sand, and (**c**) clay components in 96 natural fault cores obtained from the several fault zones in South Korea. These particle-size data were used to determine the mixing ratios of constituent components in the artificial specimens.

Results of the particle-size analysis showed that breccia constituted 0–45 wt.% of the total material, mainly 0–5 wt.% (Fig. 4a). Sand and clay contents varied from 0 to 100 wt.%, but mainly 45–65 and 30–35 wt.%, respectively (Fig. 4b,c). On the basis of these results, the component mixing ratios for the artificial specimens were determined in terms of a 20 wt.% interval with ranges of 0–40 wt.% breccia, 0–60 wt.% sand, and 20–100 wt.% clay, with 11 types of specimen consequently being manufactured (Table 2).

**Table 2:** Mixing ratios of constituent components used in each artificial specimen type.

Specimen No	Breccia (wt.%)	Sand (wt.%)	Clay (wt.%)
S-1	0	0	100
S-2	0	20	80
S-3	0	40	60
S-4	0	60	40
S-5	20	0	80
S-6	20	20	60
S-7	20	40	40
S-8	20	60	20
S-9	40	0	60
S-10	40	20	40
S-11	40	40	20

### Manufacture of artificial specimens

**Figure 5:**
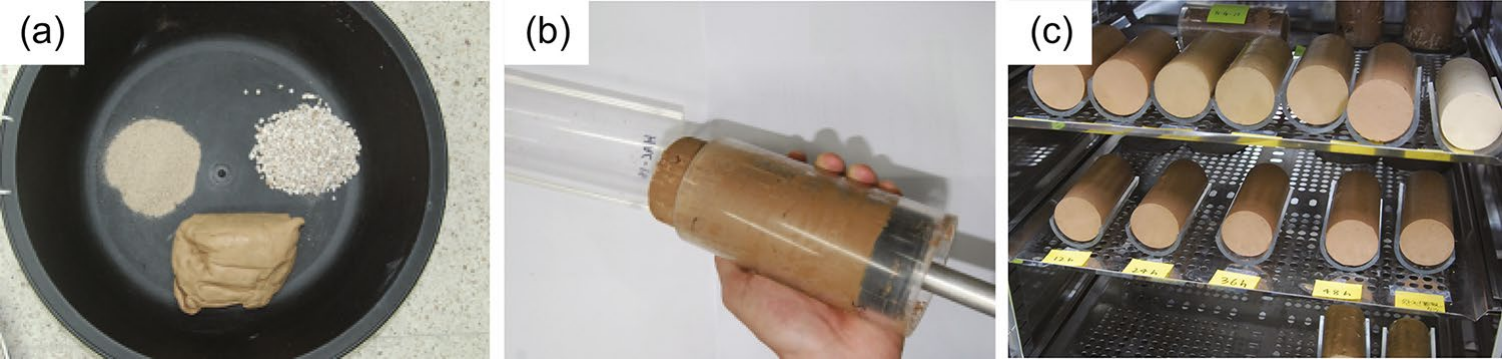
Photographs showing the preparation procedure for artificial specimens. (**a**) Constituent components (breccia, sand, and clay) ready for mixing. These materials were mixed with water according to the mixing ratios given in Table 2. (**b**) Cylindrical specimens formed using acrylic tubes. (**c**) Artificial specimens being dried at 40 ℃ for various durations (0, 12, 24, 36, 48, or 72 h) to produce specimens with different water contents.

For manufacturing the artificial specimens, breccia, sand, and clay were prepared in appropriate quantities for the mixing ratios listed in Table 2 and mixed with water (Fig. 5a). The resultant materials were compacted for > 2 h to generate a degree of cohesion/bonding within them. Artificial specimens were formed as cylinders by inserting an acrylic tube into the compacted materials for subsequent unconfined compression tests and penetration resistance tests (Fig. 5b). The diameter and length of the cylindrical specimens were 6 cm and 14 cm, respectively, following ASTM D2166-16^73^. Twelve specimens (i.e., two specimens at each of the six water contents, one for unconfined compression tests and one for penetration resistance tests) were prepared at each of the eleven mixing ratios (S-1 to S-11) listed in Table 2, giving a total of 132 specimens. Drying times were set at 0, 12, 24, 36, 48, or 72 h to produce specimens with six different water contents. Drying was performed at 40 °C to minimize the chemical change of clay minerals that might otherwise have occurred at higher temperatures (Fig. 5c).

## Experiments

### Calculation of water content

**Figure 6:**
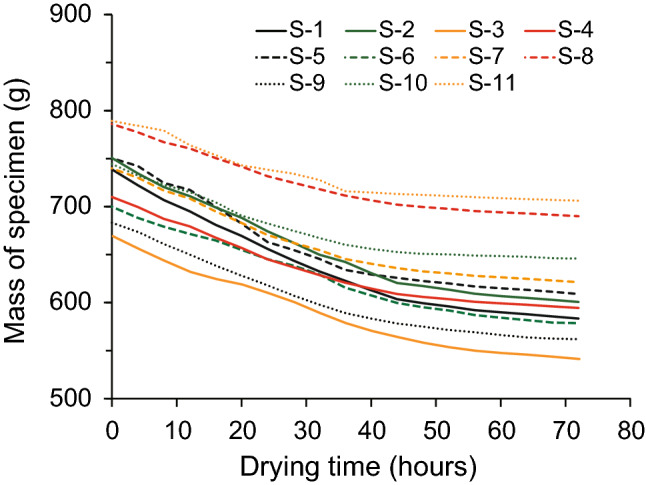
Change in mass with drying time of specimens dried for 72 h. Very little change in mass is observed after 65 h.

Water content is generally calculated from the mass of water (M_w_) of the moist specimen and the mass of the completely dried specimen (M_cds_)^74^. However, during the present study it is impossible to measure M_w_ or M_cds_ because the specimens are destroyed as a result of unconfined compression tests being conducted before complete dryness. Therefore, water contents were calculated indirectly as a function of the masses of specimens dried for 72 h. Figure 6 shows the change in mass by drying time of specimens dried for 72 h at each mixing ratio. The mass of specimens decreases rapidly during the first part of drying and becomes almost constant after ~ 65 h. We infer that the specimens were almost completely dried after 72 h of drying. Thus, the initial water content (ω_i_) of these specimens before oven drying can be calculated by Eq. (1) ^74^.

1 $${\omega }_{i}=\frac{{M}_{ms} - {M}_{cds}}{{M}_{cds}}\times 100=\frac{{M}_{w}}{{M}_{cds}}\times 100$$

where ω_i_ is the initial water content of the moist specimen before oven drying (%), M_ms_ is the mass of the moist specimen before oven drying (g), M_cds_ is the mass of a completely dried specimen (after 72 h of drying time) (g), and M_w_ is the total mass of water within a moist specimen calculated from M_ms_ and M_cds_ (g).

Furthermore, each ω_i_ of these specimens should be equal to those of other specimens (dried for 0, 12, 24, 36, or 48 h) with the same constituent component ratio because they were manufactured under the same conditions. Thus, the M_w_ of the specimens (dried for 0, 12, 24, 36, or 48 h) can be calculated by Eq. (3), which is derived from Eqs. (1) and (2).

2 $${M}_{cds}={M}_{ms}-{M}_{w}$$

3 $${M}_{w}=\frac{{M}_{ms} \times {\omega }_{i}}{{1 + \omega }_{i}}$$

where the M_w_ of each specimen (dried for 0, 12, 24, 36, or 48 h) is calculated from ω_i_ of a specimen dried for 72 h with the same proportions of constituent components.

Consequently, the water content (ω) of each specimen was calculated from Eq. (4) by measuring its mass before and after drying, and these water contents are given in Table 3.

4 $$\omega =\frac{{M}_{wd}}{{M}_{cds}}\times 100=\frac{{M}_{ms} - {M}_{ds}}{{M}_{ms} - {M}_{w}}\times 100$$

where ω is the water content of an oven-dried specimen (Δ%), M_wd_ is the mass of remaining water within an oven-dried specimen (g), and M_ds_ is the mass of an oven-dried specimen (g).

**Table 3:** Water contents of specimens according to drying time.

Specimen no	Water content (ω, Δ%)					
	0 h (a)	12 h (b)	24 h (c)	36 h (d)	48 h (e)	72 h (f)
S-1	26.7	24.9	21.6	14.6	5.0	0.0
S-2	22.7	21.3	18.6	13.2	2.7	0.0
S-3	23.5	18.6	12.6	9.5	4.7	0.0
S-4	19.4	14.1	9.7	4.5	1.4	0.0
S-5	23.0	21.7	18.8	13.2	3.5	0.0
S-6	21.0	17.0	11.3	6.8	3.2	0.0
S-7	19.1	13.8	7.7	4.8	1.7	0.0
S-8	13.9	11.1	3.4	2.0	1.5	0.0
S-9	21.6	15.1	10.2	5.6	3.0	0.0
S-10	15.2	10.7	5.7	2.6	1.4	0.0
S-11	11.8	7.1	3.0	1.6	1.0	0.0

Drying times are as indicated, varying between 0 and 72 h, with specimen sets being denoted by (a) to (f), respectively. As the water content (ω) at each drying interval was calculated from the specimen dried for 72 h, water contents were expressed as the difference (Δ%) with the water content of that specimen.

### Unconfined compression tests

Unconfined compression tests were conducted to determine the UCS and E of each specimen. Tests were conducted using equipment with a maximum load capacity of 50 kN. Loads were applied at a fixed rate of displacement of 1.0 mm/min. Axial strain and load were recorded at 0.2 s intervals, and loading continued until the compressive stress decreased after failure or the strain reached 15%^73^. Values of UCS and E (secant modulus at 50% ultimate strength (E_s50_) and tangent modulus at 50% ultimate strength (E_t50_)) were determined from the measured stress–strain curves following International Society for Rock Mechanics and Rock Engineering^75^.

**Figure 7:**
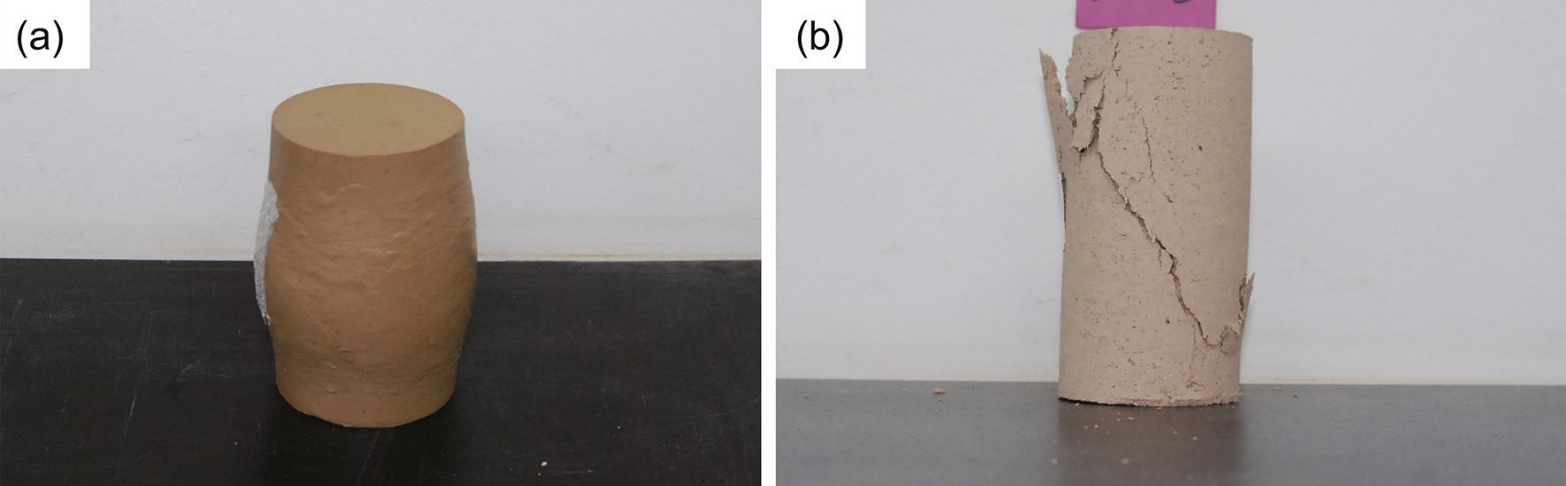
Photographs of (**a**) S-2a and (**b**) S-11d after unconfined compression tests. S-2a was deformed plastically, without any failure plane, whereas S-11d shows a failure mode typical of rock.

**Figure 8:**
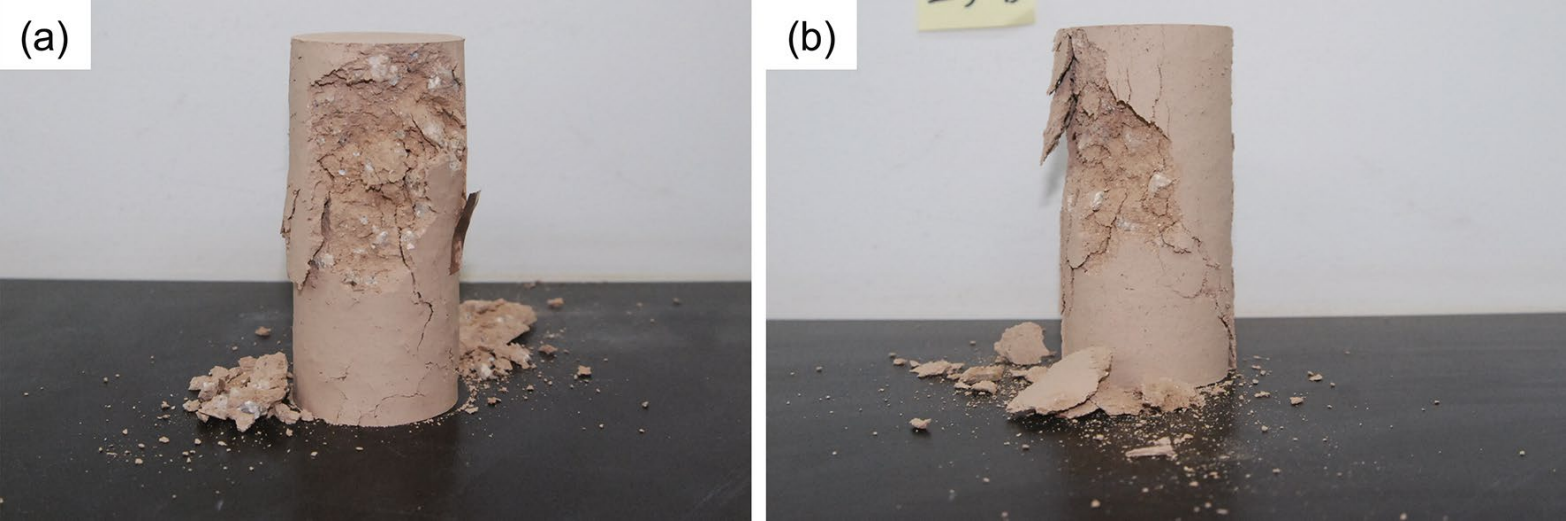
Photographs of failure planes in (**a**) S-6E and (**b**) S-7d passing around breccia after unconfined compression tests.

Unconfined compression test results show that failure did not occur in specimen sets (a) or (b), which have higher water contents compared with other specimens, with only plastic deformation being observed (Fig. 7a). In contrast, specimen sets (c) to (f), with higher water contents, showed a failure mode similar to that of rock (Fig. 7b). Failure planes for specimens containing breccia occurred mostly along breccia boundaries (Fig. 8). Such failure planes typically appear when either the breccia–matrix contact is weak or the matrix itself is weak^14,17^.

**Figure 9:**
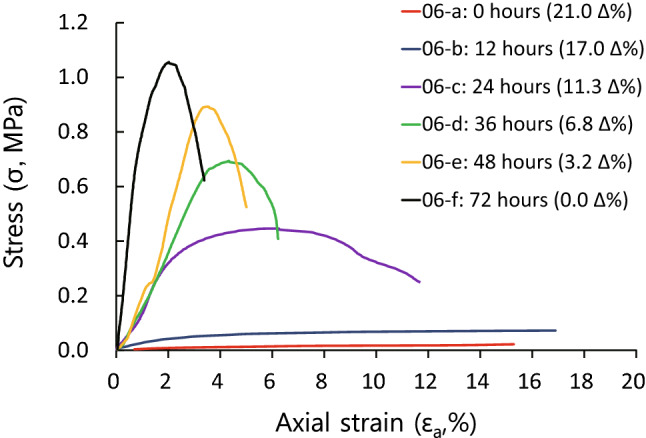
Stress–strain curves from unconfined compression tests performed on specimen set S-6. Both UCS and slope (E) in the stress–strain curves increase as water content decreases (from S-6a to S-6f). Specimens S-6a and 6b show continuous creep behavior as strain increases. Specimen S-6f shows elastic–plastic deformation and specimens S-6c–e show plastic–elastic–plastic deformation. The numbers in parentheses indicate the water content for each sample after a given drying time.

Typical stress–strain curves (for specimen set S-6) for each water content (drying time) are shown in Fig. 9. Both UCS and slope (E) in the stress–strain curves increase as water content decreases (from S-6a to S-6f). Also, specimens S-6a–b, which have higher water contents compared with other specimens, show continuous creep behavior as strain increases. In contrast, specimen S-6f, with the lowest water content, shows elastic–plastic deformation with increasing strain. Specimens S-6c–e show plastic–elastic–plastic deformation^76^. Values of UCS and E of each tested specimen, as determined from the stress–strain curves, are given in Table 4.

**Table 4:** Values of UCS, E_s50_, and E_t50_ determined from stress–strain curves for each specimen. E_s50_ is the secant modulus at 50% ultimate strength, and E_t50_ is the tangent modulus at 50% ultimate strength.

Specimen no.	UCS (MPa)						E_s50_ (MPa)						E_t50_ (MPa)					
	a	b	c	d	e	f	a	b	c	d	e	f	a	b	c	d	e	f
S-1	0.06	0.08	0.18	0.91	2.67	2.79	1.48	2.95	6.29	35.77	75.66	107.55	0.52	0.94	4.50	23.46	96.50	200.31
S-2	0.03	0.04	0.10	0.60	2.09	2.65	2.69	2.70	3.68	24.03	61.13	115.30	1.52	1.26	2.02	46.91	91.91	146.49
S-3	0.01	0.04	0.30	0.66	1.29	1.25	0.09	0.95	8.56	19.91	36.70	24.44	0.09	0.99	6.83	24.47	49.26	48.02
S-4	0.01	0.12	0.24	0.61	0.75	0.94	0.21	3.36	17.23	57.37	41.71	35.71	0.17	1.81	11.66	47.41	46.59	47.07
S-5	0.04	0.04	0.08	0.41	0.35	0.46	1.93	1.34	1.97	12.87	14.82	33.12	0.30	0.38	1.03	6.73	24.47	49.49
S-6	0.02	0.08	0.48	0.73	0.93	1.08	0.37	1.81	16.31	17.73	23.30	93.31	0.22	1.02	16.09	23.94	96.43	97.75
S-7	0.01	0.11	0.42	1.09	0.90	1.27	0.27	3.78	12.49	24.90	45.99	87.55	0.16	3.36	15.39	45.06	15.74	97.81
S-8	0.03	0.08	0.26	0.36	0.44	0.58	0.26	3.00	13.73	16.56	32.32	79.81	0.20	2.18	9.33	9.08	47.18	36.56
S-9	0.02	0.12	0.31	0.27	0.22	0.33	0.30	5.56	7.18	5.47	6.74	9.01	0.12	3.61	15.37	6.78	3.89	22.67
S-10	0.03	0.19	0.48	0.55	0.40	0.47	0.45	11.26	25.60	27.88	28.52	32.09	0.31	16.04	23.53	24.05	48.72	23.08
S-11	0.03	0.15	0.40	0.43	0.83	0.96	0.22	6.47	39.16	60.41	104.18	141.96	0.37	7.84	47.60	96.17	143.14	147.09

### Penetration resistance tests

Penetration resistance tests were conducted on the artificial specimens to establish whether this simpler test could be used as a proxy for unconfined compression testing of natural fault core, which, as mentioned, is challenging in terms of specimen sampling and preparation. The penetration resistance test is also less time-consuming and less costly than unconfined compression testing and is a non-destructive testing technique that is independent of specimen shape^48,77–79^. In particular, the NPT, which was developed by Maruto Testing Machine Company^80^ and is widely used in engineering projects, has been approved as an ISRM suggested method and can be applied to soft rock with UCS < 10 MPa^20,79,81–83^. Previous studies have used the NPT to estimate physico-mechanical properties of soft, low-strength rocks^19,20,47,49–51,84^.

**Figure 10:**
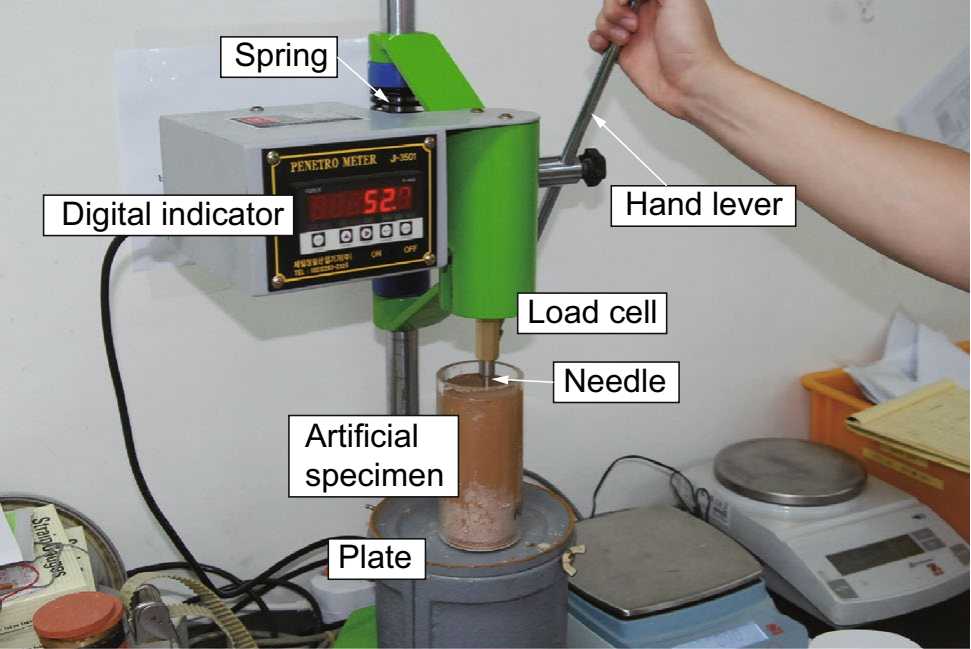
Penetrometer with digital output used for penetration resistance tests.

Penetration resistance tests conducted during the present study followed test methods ASTM C403/C403M-16^85^ and ASTM C803/C803M-18^86^, which are used to estimate the in-place strength of concrete or mortar and assess the effects of variables such as water content, using probes or pins. The test apparatus used was a hydraulic digital penetrometer that measures a maximum PRV of 1000 N and has an accuracy of ± 1% (Fig. 10). The probe is made of shaft-shaped hardened steel and has a diameter of 6.35 mm.

Penetration resistance tests were performed on specimens having the same mixture component ratios and water contents as those of specimens used in the unconfined compression tests. For the tests, the probe was slowly inserted into the specimen until the penetration depth reached 25.4 mm. During this insertion, the maximum value of penetration load was recorded from the digital display, following which the probe was extracted. This process was repeated five times at different sites on each specimen, and the mean value of these readings for each specimen was adopted as the indicative value. Values for some specimens were excluded because fracturing occurred during the penetration procedure, mostly for samples with lower water contents. PRV (in MPa) was calculated as follows:

5 $$\mathrm{PRV}={F}_{PL}/{A}_{N}$$

where F_PL_ is the penetration load value (N; i.e., the mean value of the five maximum load values read from five penetration tests on each specimen) and A_N_ is the area (0.317 cm^2^) of the probe. PRVs of the tested specimens are reported in Table 5.

**Table 5:** Penetration resistance values determined for the tested specimens. Missing values are from specimens in which fracturing occurred during penetration testing.

Specimen no.	Penetration resistance value (MPa)					
	a	b	c	d	e	f
S-1	1.69	1.96	6.64	6.80	7.52	–
S-2	0.50	0.66	0.88	5.85	–	
S-3	0.14	0.67	3.09	–		–
S-4	0.15	1.64	9.27	–	–	–
S-5	0.49	0.49	2.28	7.13	17.11	
S-6	0.50	7.46	9.67	12.52	15.16	–
S-7	0.31	3.06	6.77	11.06	11.40	–
S-8	0.99	4.31	–	–	–	–
S-9	0.70	4.67	10.76	12.76	15.64	–
S-10	8.08	11.70	19.09	27.37	–	–
S-11	1.23	21.90	32.20	36.55	37.29	–

## Results and discussion

### Effect of breccia content on the mechanical properties of fault core

The presence of breccia (or fragments) characterizes the mechanical properties of a multi-component mixture such as fault core^38,87^. The experimental results reported in Tables 2 and 4, showing the relationships between breccia content and UCS, E_s50_, and E_t50_ were used in the analysis of the studied artificial fault cores.

#### Effect of breccia content on UCS

**Figure 11:**
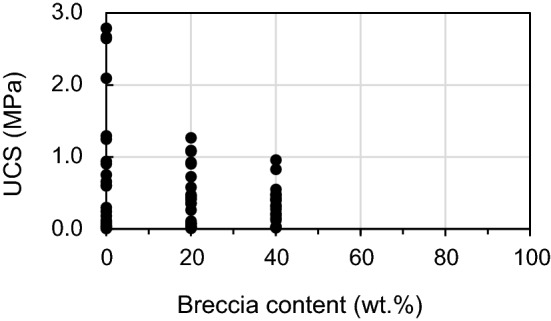
Effect of breccia content on the unconfined compressive strength (UCS) of artificial fault core. The range of UCS depends on the breccia content.

UCS values are 0.01–2.79, 0.01–1.27, and 0.02–0.96 MPa for breccia contents of 0, 20 and 40 wt.%, respectively, showing that the UCS-value decreases when breccia content increases (Table 6, Fig. 11). The mean values of UCS decrease from 0.77 to 0.43 to 0.34 MPa as breccia content increases from 0 to 20 to 40 wt.%, respectively (Table 6). This pattern is consistent with the experimental results for fault rocks reported by Medley^39^ and Kahraman and Alber^14^, but differs from those reported by Sonmez et al.^88^, who found increased UCS with increasing breccia content. Kahraman and Alber^14^ explained these different relationships in terms of the relative strengths of breccia and matrix in the fault rocks, with fault breccia (Ankara agglomerate) being stronger than matrix in the samples of Sonmez et al.^88^, and conversely in the samples of Kahraman and Alber^14^, where the fault breccia was composed of shale. However, in the present study the fault breccia is composed of granite and is therefore stronger than the clay-based matrix, meaning that the results shown in Fig. 11 cannot be explained in terms of breccia/matrix strength contrasts. Kobayashi et al.^89^ interpreted the observed decrease in strength with increasing fragment content in their artificial specimens composed of gravelly soft rock in terms of a non-uniform stress distribution and local yielding within specimens. Lindquist and Goodman^38^ reported a proportional relationship between breccia content and strength of mélanges when the volumetric proportion of breccia was relatively high. We thus explain the observed decrease in UCS with increasing breccia content in our artificial specimens in terms of the low volumetric proportion of fault breccia and the weak cohesion/bonding between the different components of the unconsolidated fault core materials.

**Table 6:** UCS values of artificial fault cores for breccia contents of 0, 20, and 40 wt.%.

Parameter		Breccia content (wt.%)		
		0	20	40
Number of specimens		24	24	18
UCS value (MPa)	Minimum	0.01	0.01	0.02
	Maximum	2.79	1.27	0.96
	Mean	0.77	0.43	0.34
	Standard deviation	0.89	0.38	0.25

#### Effect of breccia content on elastic modulus (E_s50_ and E_t50_)

**Figure 12:**
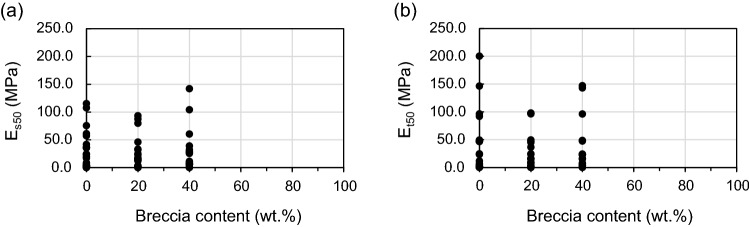
Effect of breccia content on (**a**) the secant modulus at 50% ultimate strength (E_s50_) and (**b**) the tangent modulus at 50% ultimate strength (E_t50_) of artificial fault-core-zone materials.

Maximum, minimum, and mean values of E_s50_ and E_t50_ with respect to breccia content are given in Table 7. The ranges of E_s50_ are 0.1–115.3, 0.3–93.3, and 0.2–142.0 MPa for breccia contents of 0, 20, and 40 wt.%, respectively, and the respective mean values of E_s50_ are 28.6, 22.5, and 28.5 MPa. The range and mean value of E_s50_ for breccia contents of 0 and 40 wt.% are similar in each case, and higher than those for 20 wt.% (Table 7, Fig. 12a). The range and mean value of E_t50_ with respect to breccia content are the same as those for E_s50_ (Table 7, Fig. 12b). The relationship between E and breccia content is more complex than that between UCS and breccia content, and is more difficult to explain. This means that the strain occurring until the specimen reaches its yield strength is also affected by other factors besides breccia. Each specimen plotted in Fig. 12 contains sand and clay mixed in various ratios, but these were excluded from the analysis and only breccia content was considered as a variable. For a heterogeneous material such as a fault core, the local stress or strain is not uniform since the stress is concentrated at the interface between the constituent materials (eg, the boundary between breccia and clay)^90^. That is, the bonding or behavior between the particles may vary depending on the composition ratio of these constituent materials due to the difference in properties between the breccia and the surrounding matrix (sand or clay). For this reason, the distribution of the constituent materials (grain size in this study) may be a factor influencing the strain when stress is applied to the specimens.

**Table 7:** Elastic modulus (E_s50_ and E_t50_) of artificial fault core for breccia contents of 0, 20, and 40 wt.%.

Parameter		Breccia content (wt.%)		
		0	20	40
Number of specimens		24	24	18
E_s50_ (MPa)	Minimum	0.1	0.3	0.2
	Maximum	115.3	93.3	142.0
	Mean	28.6	22.5	28.5
	Standard deviation	32.7	27.1	37.5
E_t50_ (MPa)	Minimum	0.1	0.2	0.1
	Maximum	200.3	97.8	147.1
	Mean	37.5	25.0	35.0
	Standard deviation	50.2	31.4	45.2

**Figure 13:**
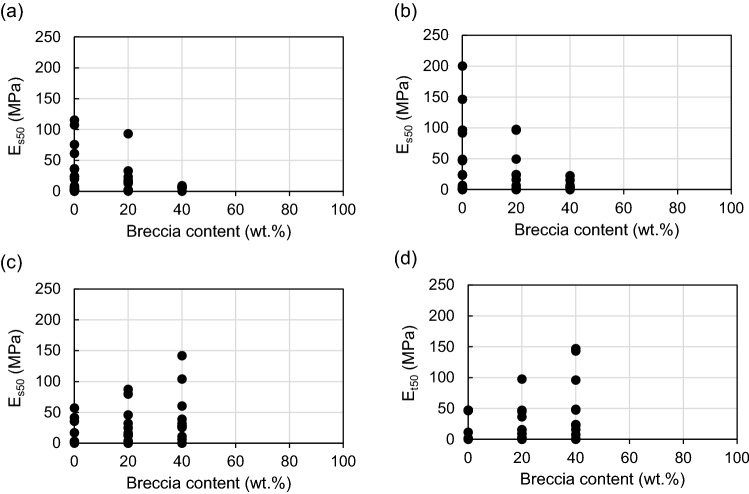
Relationship between E (E_s50_ and E_t50_) and breccia content classified based on clay contents of 50 wt.%. **(a)** E_s50_ and **(b)** E_t50_ show a proportional relationship with the breccia content in specimens for clay content of ≥ 50 wt.%. **(c)** E_s50_ and **(d)** E_t50_ show an inverse relationship with breccia content for clay contents of < 50 wt.%.

Figure 13 shows the relationship between E and breccia content after classifying the specimens based on the clay content of 50 wt.%. According to the USCS^70^, it is divided into fine-grained (clay ≥ 50 wt.%) and coarse-grained (clay < 50 wt.%) based on the clay content of 50 wt.%, which is the most common indicator of the particle size characteristics of materials. As a result, the relationship between E and breccia content for clay contents of ≥ 50 wt.% was inversely proportional relationship similar to that between UCS and breccia content (Fig. 13a,b). In contrast, the relationship between E and breccia content for clay contents of < 50 wt.% shows a proportional relationship (Fig. 13c,d). Here, it can be seen that specimens (S-1, 2, 3, 5, 6 and 9) with clay content ≥ 50 wt.% were consisted of only one or two materials (only clay/sand and clay/breccia) except for S-6. That is, in specimens with relatively simple constituents, an increase in breccia content means an increase in the interface between the constituents, which is considered to be the cause of the decrease in E as shown in Fig. 13a,b. In contrast, specimens (S-1, 2, 3, 5, 6 and 9) with clay content ≥ 50 wt.% contained all of breccia, sand and clay except for S-4. That is, Fig. 13c,d shows that the presence of breccia within specimens with various particle sizes is a factor that produces less strain according to stress. However, since the artificial specimens in this study have different water content, there is a limit to simply discussing the relationship between breccia and E with only the particle distribution. For this reason, Kahraman and Alber^14^ also argued that more investigations and data are needed to clarify the relationship between breccia content and E. Therefore, in this study, in order to approach the cause more closely, an analysis that additionally reflected the water content was conducted.

### Effect of water content on the mechanical properties of fault core

**Figure 14:**
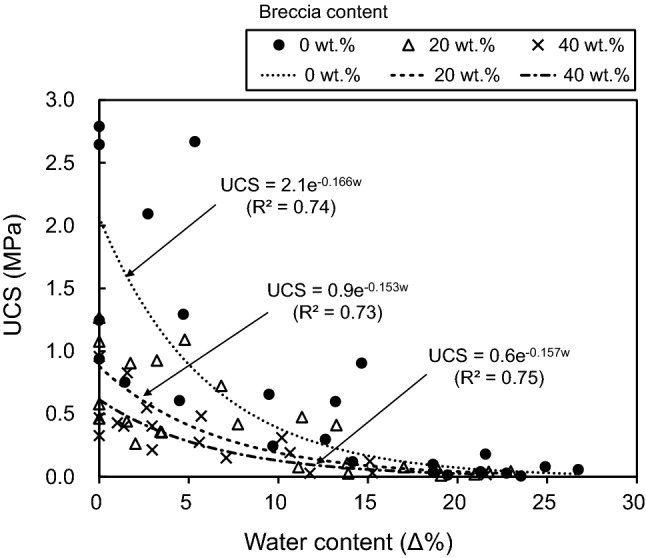
Relationship between unconfined compressive strength (UCS) and water content with respect to three breccia contents (0, 20, and 40 wt.%). The regression models show that UCS is exponentially related to water content for distinct breccia contents of 0, 20, and 40 wt.%.

The effect of water content on the mechanical properties of the artificial fault core was examined by performing correlation analysis between water content and UCS, E_s50_, and E_t50_ while holding breccia content constant. Increasing water content is known to reduce material strength^21,44,48,91,92^. For example, Avşar et al.^20^ identified a decrease in UCS with increasing water content for water contents of > 11% in weakly bonded volcanic soils. This pattern was explained by a reduction in the degree of interlocking between grains caused by higher proportions of water, but only above a threshold water content (11%). Here, the UCS-value for the artificial specimens shows a decrease as a function of increasing water content with marked reductions in UCS occurring between water contents of 0 Δ% and 5 Δ% (Fig. 14). In addition, for similar water content, specimens with high breccia contents have lower UCS values. Data are scattered for a water content of 0 Δ% and for a breccia content of 0 wt.%. Still, regression show that UCS is exponentially related to water content for distinct breccia contents of 0, 20, and 40 wt.%. The coefficients of determination (R^2^) of 0.74, 0.73, and 0.75, respectively (Fig. 14). These are high correlations considering the heterogeneity and anisotropy of the fault cores. The relationship between water content and UCS, expressed as an exponential function, is similar to that found previously for soft rocks^21,93,94^. In addition, Erguler and Ulusay^48^ and Hawkins^91^ reported that the main reduction in strength in stronger rocks occurs between water contents of 0% and 2%.

**Figure 15:**
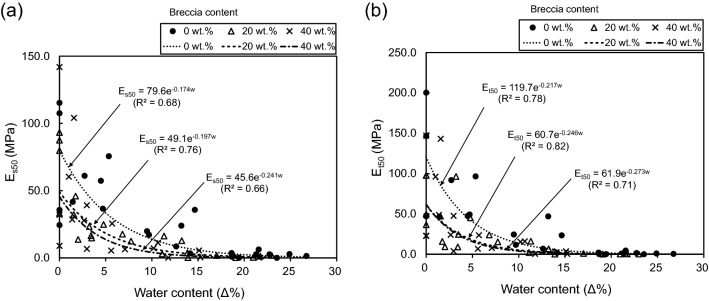
Relationships between (**a**) the secant modulus at 50% ultimate strength (E_s50_) and (**b**) the tangent modulus at 50% ultimate strength (E_t50_) and water content with respect to three breccia contents (0, 20, and 40 wt.%). The relationships between E_s50_ or E_t50_ are inversely related to water content.

The E_s50_ or E_t50_ are inversely related to water content (Fig. 15), similar to UCS. R^2^ values for E_s50_ and water content are 0.68, 0.76, and 0.66 for breccia contents of 0, 20, and 40 wt.%, respectively (Fig. 15a), and for E_t50_ and water content are 0.78, 0.82, and 0.71 for these breccia contents, respectively (Fig. 15b), with both showing the highest correlation at a breccia content of 20%.

In summary, both UCS and E of the artificial fault cores decrease with increasing water content, and these mechanical properties show high correlations with water content for each value of breccia content. The equations describing the relationships between parameters calculated from regression analysis are reported in Table 8.

**Table 8:** Correlations between water content and mechanical properties (UCS, E_s50_, and E_t50_) for breccia contents of 0, 20, and 40 wt.%.

Mechanical properties	Breccia content (wt.%)	Regression coefficients	Estimate	P-value	Adjusted R-squared	Regression equations
UCS (MPa)	0	Intercept	2.064	0.000***	0.747	${\text{UCS}}={2.1e}^{-0.166\omega }$
		Water content	−0.166	0.004**		
	20	Intercept	0.880	0.000***	0.725	${\text{UCS}}={0.9e}^{-0.153\omega }$
		Water content	−0.153	0.000***		
	40	Intercept	0.616	0.000***	0.751	${\text{UCS}}={0.6e}^{-0.157\omega }$
		Water content	−0.157	0.000***		
E_S50_ (MPa)	0	Intercept	79.6	0.000***	0.683	${\text{UCS}}={79.6e}^{-0.174\omega }$
		Water content	−0.174	0.015*		
	20	Intercept	49.1	0.000***	0.764	${\text{UCS}}={49.1e}^{-0.197\omega }$
		Water content	−0.197	0.002**		
	40	Intercept	45.6	0.000***	0.664	${\text{UCS}}={45.6e}^{-0.241\omega }$
		Water content	−0.241	0.018*		
E_t50_ (MPa)	0	Intercept	119.7	0.000***	0.780	${\text{UCS}}={119.7e}^{-0.217\omega }$
		Water content	−0.217	0.011*		
	20	Intercept	60.7	0.000***	0.818	${\text{UCS}}={60.7e}^{-0.246\omega }$
		Water content	−0.246	0.003**		
	40	Intercept	61.9	0.000***	0.710	${\text{UCS}}={61.9 e}^{-0.273\omega }$
		Water content	−0.273	0.020*		

R^2^ is the coefficient of determination, and ω is water content (Δ%).

^※^P-value: 0 < *** < 0.001 < ** < 0.01 < * < 0.05 < . < 0.1 <  < 1 (significant: < 0.05).

### Correlations between mechanical properties

#### Estimation of UCS using PRV

**Figure 16:**
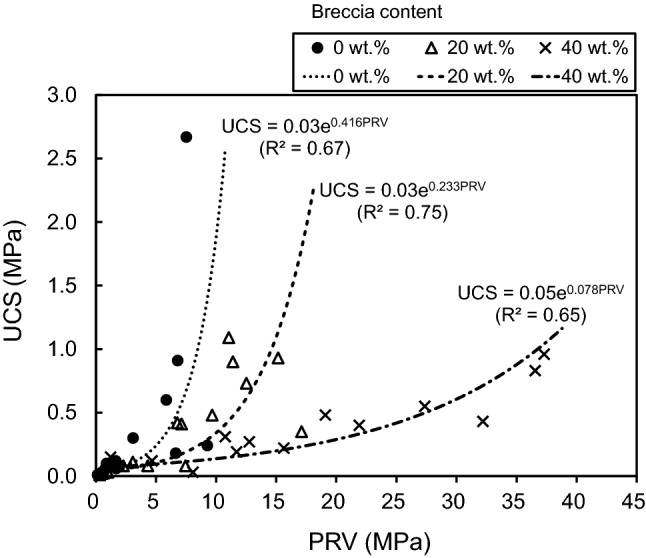
Correlation between unconfined compressive strength (UCS) and penetration resistance value (PRV) with respect to three levels of breccia content (0, 20, and 40 wt.%).

PRV might be a useful parameter for estimating the UCS of natural fault cores using a portable penetrometer in the field, and particularly so because such materials are difficult to sample, and because it impossible to preserve the in-site sample configuration when sampling. Furthermore, preparation for unconfined compression testing is challenging. Generally, a positive correlation exists between PRV and UCS is common^20,95^, and is supported by results of this study (Fig. 16). Erguler and Ulusay^48^ found that correlation between needle penetration resistance and UCS is uncertain in low-strength (< 5 MPa) clay-bearing rock, making UCS is difficult to predict. We find, however, that PRV and UCS in the studied artificial specimens are proportional to each other and have a different relationship for each of the three levels of breccia content when UCS-values are < 3 MPa (Fig. 16). In addition, for a given UCS-value, the PRV increases with breccia content, which indicates that although PRV and UCS show a moderately strong relationship, the nature of the relationship varies with the constituent components of the material. Therefore, to determine the UCS using PRV it is necessary to consider additional variables such as the breccia content, as done here, rather than taking a simplistic approach.

#### Relationship between elastic modulus (E_t50_ and E_s50_) and UCS

**Figure 17:**
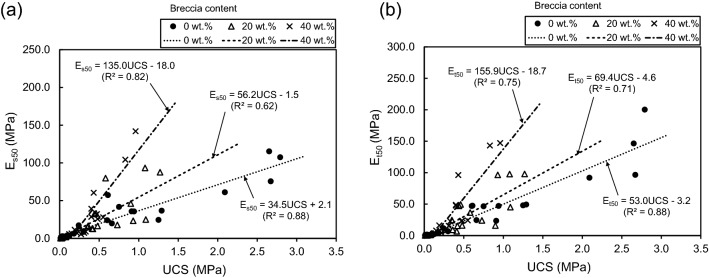
Relationships between (**a**) the secant modulus at 50% ultimate strength (E_s50_) and (**b**) the tangent modulus at 50% ultimate strength (E_t50_) and unconfined compressive strength with respect to three levels of breccia content (0, 20, and 40 wt.%).

Our investigations of artificial specimen show that E is proportional to UCS at each breccia content (Fig. 17). R^2^ values for E_s50_ and UCS are 0.88, 0.62, and 0.82 for breccia contents of 0, 20, and 40 wt.%, respectively (Fig. 17a), and for E_t50_ and UCS are 0.88, 0.71, and 0.75, respectively (Fig. 17b), showing the lowest correlation at a breccia content of 20 wt.%. In addition, for similar UCS, specimens with higher breccia contents have higher E, suggesting that low strain results from the presence of breccia. These results indicate that the E value of fault core is proportional to the strength of the component itself, unlike the UCS, which is analyzed in terms of the bonding between constituent components.

**Figure 18:**
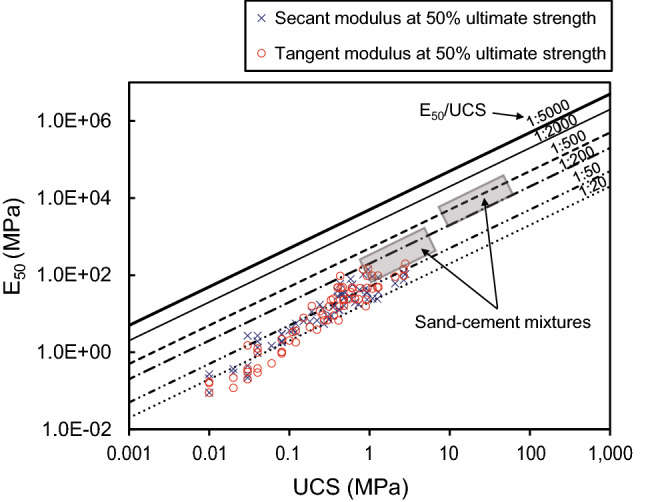
Relationship between elastic modulus (E_t50_ and E_s50_) and unconfined compressive strength (UCS) for the studied artificial fault cores, with data for soft rocks (ratio lines) and sand–cement mixtures (shaded rectangles) shown for comparison. The artificial fault cores with UCS < 0.1 MPa are located mostly below the 1:50 ratio line, and those with UCS > 0.1 MPa lie mostly between the 1:20 and 1:200 ratio lines. The E_50_/UCS lines and shaded sand–cement data are from Kanji^21^, modified after Kanji and Galván^92^, Galván^44^, and Deere^101^.

Correlations between UCS and E have been previously reported in experiments involving soft rocks and sand–cement mixtures^91,96–100^. Galván^44^ analyzed data collated from numerous studies and on that basis plotted the relationship (ratio) between UCS and E_50_ (Fig. 18). The E_50_/UCS ratio was originally proposed by Deere^101^ and has since been used for basic descriptions of rock mechanical properties^21^. Figure 18 show that most of results are consistent with the properties of natural rocks. The artificial fault cores have lower UCS and E than sand–cement mixtures. In particular, the data of fault cores and sand–cement mixtures have similar slopes in the figure. These results indicate that the characteristics of the two artificially made specimens are similar to each other and that the clay materials have lower mechanical properties than sand.

## Conclusions

Since the fault core generally has a very weak strength, it is very difficult to prepare a test for estimating the mechanical properties. Therefore, if we find a factor that affects the mechanical properties and suggest a method for indirectly estimating the value, it will be helpful to engineers in the construction.

In this study, unconfined compression tests and penetration resistance tests were conducted on 132 artificial specimens to determine the mechanical properties of fault core. The specimens were manufactured by mixing breccia, sand, clay, and water, which are typical components of natural fault cores, to yield mixtures with 11 different constituent component ratios and differing water contents. The measured experimental data allowed the effects of breccia content and water content on mechanical properties (UCS, E_s50_, and E_t50_) to be determined and relationships between mechanical properties to be established. Our main conclusions are as follows.

1. UCS of the artificial fault core decreases with increasing breccia content from 0 to 20 to 40 wt.%, but elastic modulus (E_s50_ and E_t50_) decreases from breccia contents of 0 to 20 wt.% and then increases to a breccia content of 40 wt.%.
2. UCS decreases as water content increases, and the reduction is most marked between water contents of 0% and 5%. Specimens with high breccia content but similar water content have lower UCS values than do those with low breccia content. Elastic modulus (E_s50_ and E_t50_) also decreases with increasing water content. UCS, E_s50_, and E_t50_ of the fault core are exponentially related to water content, and the relationship varies with breccia content.
3. Penetration resistance value (PRV) is proportional to UCS and shows different relationships and correlation strengths depending on breccia content. For a given UCS, PRV increases as breccia content increases, revealing that the nature of the relationship varies with the constituent components of the fault core.
4. Elastic modulus is proportional to UCS, and specimens with higher breccia contents have higher E_s50_ and E_t50_ for similar UCS. The UCS and E of the studied artificial fault cores are consistent with those of soft rock and sand–cement mixtures. In addition, the measured E_50_/UCS ratio shows a similar slope to that of sand–cement mixtures, although with lower values.
5. Breccia content, water content, and PRV values can be obtained through relatively simple specimen preparation and testing procedures. The results of this study should be of use for estimating the mechanical properties of fault core, which is typically highly challenging with regard to field sampling and specimen preparation for laboratory testing.

## Data Availability

The data that support the findings of this study are available from the corresponding author upon reasonable request.
